# Anatomical classification of breast sentinel lymph nodes using computed tomography–lymphography

**DOI:** 10.1007/s12565-018-0441-2

**Published:** 2018-05-03

**Authors:** Tamaki Fujita, Hiroyuki Miura, Hiroko Seino, Shuichi Ono, Takashi Nishi, Akimasa Nishimura, Kenichi Hakamada, Masahiko Aoki

**Affiliations:** 1 Department of Radiology and Radiation Oncology, Graduate School of Medicine, Hirosaki University, 5 Zaifu-cho, Hirosaki-shi, Aomori, 036-8562 Japan; 2 Department of Pathology and Bioscience, Graduate School of Medicine, Hirosaki University, 5 Zaifu-cho, Hirosaki-shi, Aomori, 036-8562 Japan; 3 Department of Gastroenterological Surgery, Graduate School of Medicine, Hirosaki University, 5 Zaifu-cho, Hirosaki-shi, Aomori, 036-8562 Japan

**Keywords:** Anatomical classification, Axillary lymph node, Breast cancer, Computed tomography–lymphography, Sentinel lymph node

## Abstract

To evaluate the anatomical classification and location of breast sentinel lymph nodes, preoperative computed tomography–lymphography examinations were retrospectively reviewed for sentinel lymph nodes in 464 cases clinically diagnosed with node-negative breast cancer between July 2007 and June 2016. Anatomical classification was performed based on the numbers of lymphatic routes and sentinel lymph nodes, the flow direction of lymphatic routes, and the location of sentinel lymph nodes. Of the 464 cases reviewed, anatomical classification could be performed in 434 (93.5 %). The largest number of cases showed single route/single sentinel lymph node (*n* = 296, 68.2 %), followed by multiple routes/multiple sentinel lymph nodes (*n* = 59, 13.6 %), single route/multiple sentinel lymph nodes (*n* = 53, 12.2 %), and multiple routes/single sentinel lymph node (*n* = 26, 6.0 %). Classification based on the flow direction of lymphatic routes showed that 429 cases (98.8 %) had outward flow on the superficial fascia toward axillary lymph nodes, whereas classification based on the height of sentinel lymph nodes showed that 323 cases (74.4 %) belonged to the upper pectoral group of axillary lymph nodes. There was wide variation in the number of lymphatic routes and their branching patterns and in the number, location, and direction of flow of sentinel lymph nodes. It is clinically very important to preoperatively understand the anatomical morphology of lymphatic routes and sentinel lymph nodes for optimal treatment of breast cancer, and computed tomography–lymphography is suitable for this purpose.

## Introduction

A sentinel lymph node (SLN) is any lymph node that directly receives lymph drainage from a tumor site (Uren et al. 2003). Uren et al. (2003) reported that SLNs need not necessarily be the nodes closest to the primary site and that lymphatic routes can bypass many other nodes before reaching SLNs.

Conventional methods for detection of SLNs include the dye method and radioisotope method. The dye method requires a high level of technical skill to trace the dye-stained lymphatic route to SLNs and can only be performed with support from a nuclear medicine department, while scintigrams obtained by the radioisotope method cannot clearly visualize the direct connection between primary SLNs and their afferent lymphatic routes (Yamamoto et al. 2016).

Computed tomography lymphography (CT-LG) involves CT after locally injecting a contrast agent. It can be conveniently performed, and the morphology and location of both lymphatic routes and SLNs can be visualized in detail, with minimal invasiveness (Yamamoto et al. 2016).

Yamamoto et al. (2016) used CT-LG to classify 549 cases into four anatomical categories, based on the numbers of lymphatic routes and lymph nodes: (a) single route/single SLN (355 cases, 65 %; single lymphatic route and lymph node), (b) single route/multiple SLNs (62 cases, 11 %; single lymphatic route with multiple lymph nodes), (c) multiple routes/single SLN (59 cases, 11 %; multiple lymphatic routes with one lymph node), and (d) multiple routes/multiple SLNs (73 cases, 13 %; multiple lymphatic routes and lymph nodes).

SLN biopsy is the gold-standard procedure for axial lymph node evaluation in breast cancer, and axillary lymph node dissection (ALND) is usually avoided in SLN-negative patients (Krag et al. 2010; The Japanese Breast Cancer Society 2016; Lyman et al. 2005, 2014). Thus, it is important to completely understand the anatomy and number of lymphatic routes and SLNs and their location. The location and flow direction of SLNs have been studied in cadavers (Suami et al. 2009a, b) and by the radioisotope method (Blumgart et al. 2011a, b), but the anatomical morphology of lymphatic routes and SLNs using CT-LG has not been extensively studied since the report of Yamamoto et al. (2016). Thus, we decided to investigate them with CT-LG.

## Patients and methods

### Patients

This retrospective study was approved by our ethics committee, and informed consent for using case information was obtained from all patients. Their anonymity is preserved.

Preoperative examinations for SLNs in 480 cases with clinically node-negative breast cancer diagnosed between July 2007 and June 2016 at our institution were retrospectively reviewed. Six cases with history of surgery and 10 with history of chemotherapy were excluded. The remaining 464 cases (100 %) were women; the patients’ characteristics are presented in Table 1.

**Table 1:** Patients’ background characteristics

Parameter	Value, *n* (%)
Number of patients	464
Age in years, average (range)	56 (24–88)
Sex	
Male	0
Female	464 (100 %)
SLN metastasis	
Positive	104 (22.4 %)
Negative	360 (77.6 %)
Histological type	
DCIS	62 (13.4 %)
Papillotubular ca.	110 (23.7 %)
Solid-tubular ca.	50 (10.8 %)
Scirrhous ca.	208 (44.8 %)
Mucinous ca.	12 (2.6 %)
Invasive micropapillary ca.	7 (1.5 %)
Invasive lobular ca.	6 (1.3 %)
Apocrine ca.	6 (1.3 %)
Medullary ca.	3 (0.6 %)

*DCIS* ductal carcinoma in situ, *ca.* carcinoma

### CT-LG procedure

CT-LG was performed using a four-detector row CT scanner (Light Speed QX/i, GE Healthcare, Milwaukee, WI, USA) until 7 February 2011, using a 64-detector row CT scanner (Somatom Definition, Siemens Healthcare, Munich, Germany) from 8 to 13 February 2011, and using a 64-detector row CT scanner (Discovery CT750 HD, GE Healthcare, Milwaukee, WI, USA) from 14 February 2011 onward, using the following parameters: 120 kV, 200–400 mA, scan time 0.5 s, and slice thickness of 1.25 mm (Light Speed QX/i), 1.00 mm (Somatom Definition), or 0.63 mm (Discovery CT750 HD). A mixture of 0.5 ml 1 % lidocaine and 1 ml iohexol (Omnipaque 300; Daiichi-Sankyo Company, Tokyo, Japan) or a mixture of 1.5 ml 1 % lidocaine and 1.5 ml iohexol was injected into the nipple, followed by gentle massage for 10 s. Each patient was placed in supine position, with arms positioned in cranial direction, as during breast cancer surgery. Scanning was initiated 60 s after contrast agent injection. Scans were appropriately added after 3 min, depending on contrast spread.

**Fig. 1:**
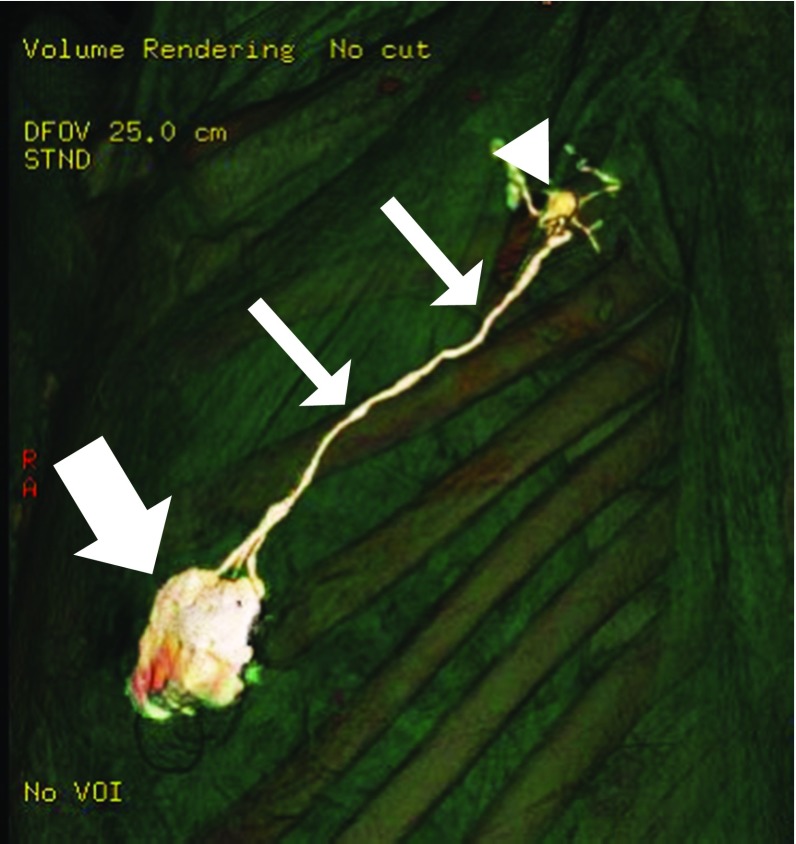
3D-CT lymphogram of woman in her 50s with left breast cancer. A single lymphatic route (thin arrow) from the periareolar area (thick arrow) drains into a single SLN (arrowhead)

All CT-LG datasets were loaded onto a workstation (Advantage Workstation VolumeShare 4 XT, GE Healthcare, Milwaukee, WI, USA) and converted into three-dimensional (3D) images (Fig. 1) using the workstation’s onboard software.

Two radiologists classified the cases based on data from the 3D images, and those with poorly visible lymphatic routes or lymph nodes were also classified using the original CT image as reference.

### Anatomical classification procedure

SLNs were first classified into four categories based on the report of Yamamoto et al. (2016), then each category was subdivided as follows: single route/single SLN was subdivided into (a) no branching, (b) breast-side branching, (c) axillary-side branching, (d) breast- and axillary-side branching, and (e) other; single route/multiple SLNs into (a) breast-side branching, (b) axillary-side branching, and (c) breast- and axillary-side branching; multiple routes/single SLN into (a) no branching in each route, (b) mixed, with and without branching, and (c) each route with separate branching; and multiple routes/multiple SLNs into (a) no branching, (b) branching on each route, and (c) branching and flow from other route.

Based on the reports of Kutsuna (1968) and Kaneko (2001), SLNs were classified into three categories, based on flow direction: (a) flowing outward on the superficial fascia toward the axillary lymph nodes, (b) flowing upward on the superficial fascia toward the infraclavicular or supraclavicular nodes, and (c) flowing toward the midline and reaching the parasternal or contralateral axillary lymph nodes.

Regional lymph nodes involved in breast cancer include the axillary, brachial, subpectoral, and infraclavicular lymph nodes. The axillary lymph nodes were further classified into (a) upper pectoral lymph nodes, (b) central axillary lymph nodes, and (c) lower pectoral lymph nodes. They were classified based on their location compared with rib level into (a) the upper pectoral lymph nodes located between the upper border of the second rib and the lower border of the third rib, (b) central axillary lymph nodes located above (a), and (c) the lower pectoral lymph nodes located between the fourth and sixth ribs.

### Statistical analysis

Possible correlations between anatomical classification (good versus poor imaging) and histological metastasis (presence versus absence) were tested by chi-squared test with 5 % significance level using Excel 2013 software (Microsoft Corporation, Redmond, WA, USA) with Statcel 4 add-in package.

## Results

Patients’ characteristics are presented in Table 1. Among the 464 patients, anatomical classification could be performed in 434 (93.5 %), but not in 30 (6.5 %). Although these 30 cases were impossible to classify, there were 7 cases where only the lymphatic routes were rendered and 4 where only the SLNs were drawn, where 19 were without rendering. Of the 434 cases that permitted anatomical classification, 99 (22.8 %) showed histological evidence of metastases, whereas 335 (77.2 %) showed no metastases. Of the 30 cases that could not be anatomically classified, 4 (13.3 %) showed histological evidence of metastases, whereas 26 (86.7 %) showed no metastases. There was no correlation between anatomical classification (good versus poor imaging) and metastases (presence versus absence) (*p* = 0.22).

**Fig. 2:**
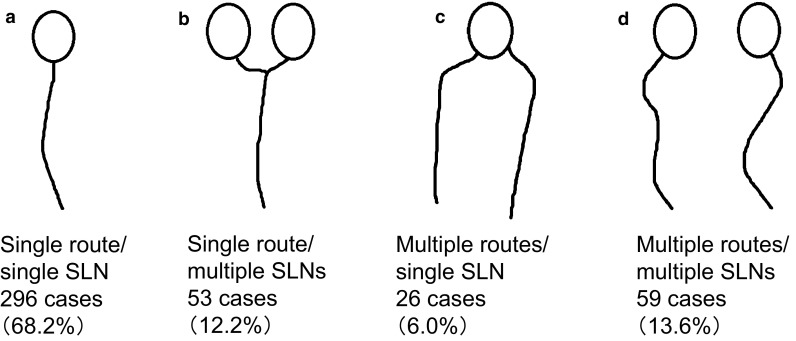
Anatomical classification of SLNs in 434 cases with reference to the report of Yamamoto et al. (2016): **a** single route/single SLN (*n* = 296, 68.2 %), **b** single route/multiple SLNs (*n* = 53, 12.2 %), **c** multiple routes/single SLN (*n* = 26, 6.0 %), and **d** multiple routes/multiple SLNs (*n* = 59, 13.6 %)

**Fig. 3:**
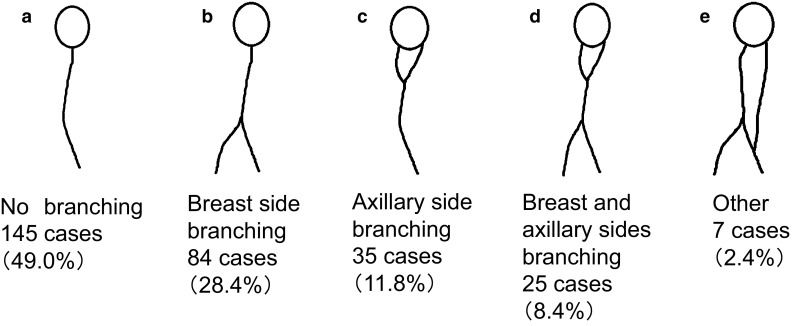
Further classification results of single route/single SLN in 296 cases: **a** no branching (*n* = 145, 49.0 %), **b** breast-side branching (*n* = 84, 28.4 %), **c** axillary-side branching (*n* = 35, 11.8 %), **d** breast- and axillary-side branching (*n* = 25, 8.4 %), and **e** other (*n* = 7, 2.4 %)

**Fig. 4:**
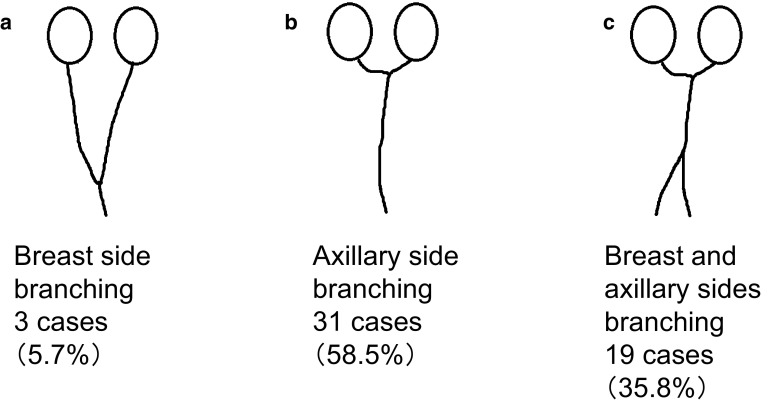
Further classification results of single route/multiple SLNs in 53 cases: **a** breast-side branching (*n* = 3, 5.7 %), **b** axillary-side branching (*n* = 31, 58.5 %), and **c** breast- and axillary-side branching (*n* = 19, 35.8 %)

**Fig. 5:**
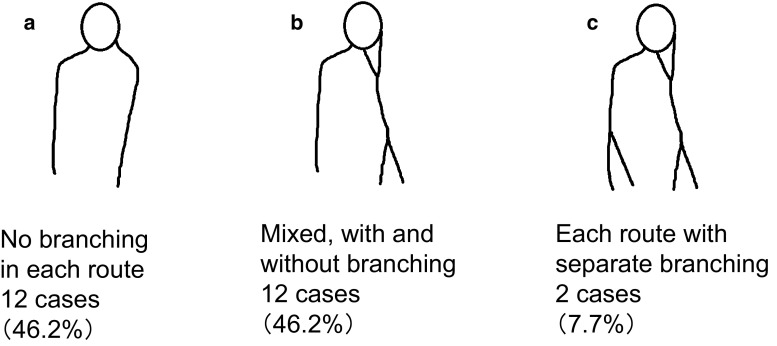
Further classification results of multiple routes/single SLN in 26 cases: **a** no branching in each route (*n* = 12, 46.2 %), **b** mixed, with and without branching (*n* = 12, 46.2 %), and **c** each route with separate branching (*n* = 2, 7.7 %)

**Fig. 6:**
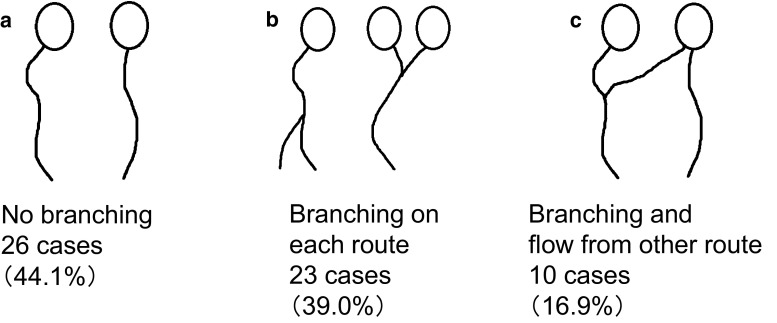
Further classification results of multiple routes/multiple SLNs in 59 cases: **a** no branching (*n* = 26, 44.1 %), **b** branching on each route (*n* = 23, 39.0 %), and **c** branching and flow from other route (*n* = 10, 16.9 %)

Among the 434 cases analyzed, the largest number of cases showed single route/single SLN (*n* = 296, 68.2 %), followed by multiple routes/multiple SLNs (*n* = 59, 13.6 %), single route/multiple SLNs (*n* = 53, 12.2 %), and multiple routes/single SLN (*n* = 26, 6.0 %) (Fig. 2). Among the 296 cases with single route/single SLN, the largest number of cases showed no branching (*n* = 145, 49.0 %) (Fig. 3). Of the 53 cases with single route/multiple SLNs, most showed axillary-side branching (*n* = 31, 58.5 %) (Fig. 4). Of the 26 cases with multiple routes/single SLN, 12 (46.2 %) showed no branching in each route, 12 (46.2 %) showed mixed, with and without branching, and 2 (7.7 %) showed each route with separate branching (Fig. 5). Of the 59 cases with multiple routes/multiple SLNs, 26 (44.1 %) showed no branching, representing the largest group (Fig. 6), whereas 4 (6.8 %) had routes that did not reach the axilla. Furthermore, branching on each route was seen in 23 cases (39.0 %), and among these, 14 (23.7 %) showed branching on either route and 9 (15.3 %) showed branching into multiple SLNs.

**Fig. 7:**
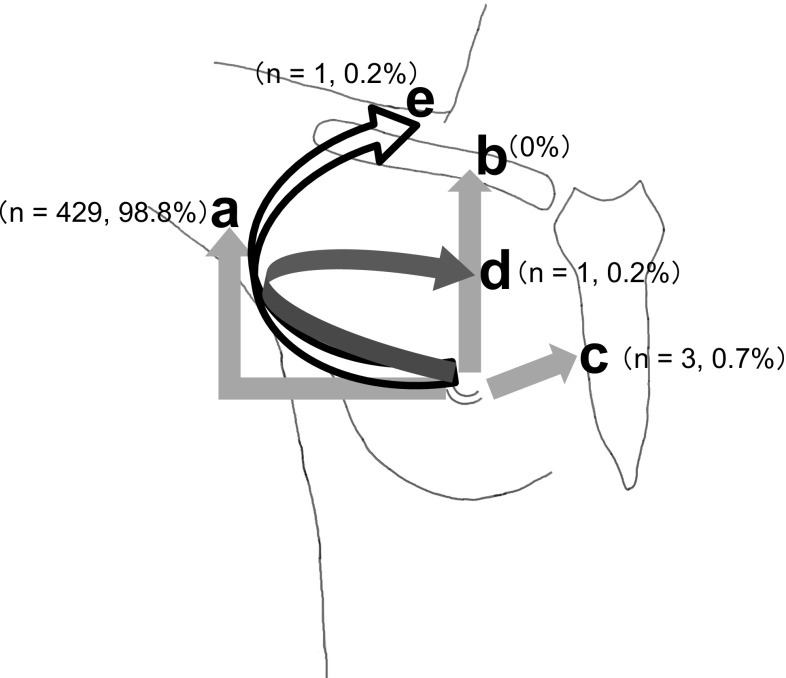
Classification results by SLN flow direction: **a** flowing outward on the superficial fascia toward the axillary lymph nodes (*n* = 429, 98.8 %), **b** flowing upward on the superficial fascia toward the infraclavicular or supraclavicular nodes (*n* = 0, 0 %), **c** flowing toward the midline and reaching the parasternal lymph nodes (*n* = 3, 0.7 %), **d** flow that reached the interpectoral lymph node via the axilla (*n* = 1, 0.2 %), and **e** flow that reached the supraclavicular lymph node via the axilla (*n* = 1, 0.2 %)

The 434 cases were also categorized according to the flow direction of SLNs, wherein 429 (98.8 %) were found to be flowing outward on the superficial fascia toward axillary lymph nodes, 3 (0.7 %) toward the midline that reached the parasternal lymph nodes, and 0 flowing upward on the superficial fascia toward the infraclavicular or supraclavicular lymph nodes (Fig. 7). Additionally, there was one case each (0.2 %) of flow that reached the interpectoral and supraclavicular lymph nodes via the axilla.

**Fig. 8:**
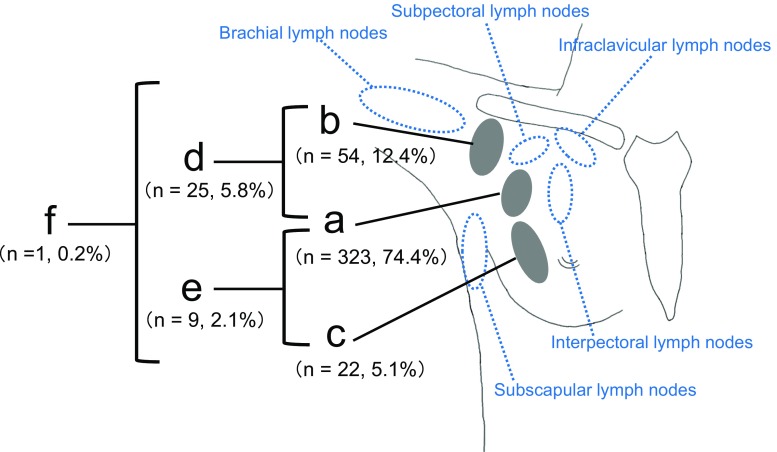
Classification results based on SLN location compared with rib level: **a** upper pectoral lymph nodes (*n* = 323, 74.4 %), **b** central axillary lymph nodes (*n* = 54, 12.4 %), **c** lower pectoral lymph nodes (*n* = 22, 5.1 %), **d** merged central axillary lymph nodes and upper pectoral lymph nodes (*n* = 25, 5.8 %), **e** merged upper and lower pectoral lymph nodes (*n* = 9, 2.1 %), and **f** both of the above-mentioned phenomena (*n* = 1, 0.2 %)

Next, all 434 cases were classified according to the rib height at which the SLNs were present; 323 (74.4 %) were upper pectoral lymph nodes, 54 (12.4 %) were central axillary lymph nodes, and 22 (5.1 %) were lower pectoral lymph nodes. In addition, there were 25 cases (5.8 %) where the central axillary lymph nodes were merged with the upper pectoral lymph nodes, 9 (2.1 %) where the upper and lower pectoral lymph nodes were merged, and 1 case (0.2 %) with both of the above-mentioned phenomena (Fig. 8).

## Discussion

The current standard of breast cancer surgery recommends ALND only if the results of SLN biopsy warrant it (Krag et al.^2010^; The Japanese Breast Cancer Society 2016; Lyman et al. 2005, 2014). Furthermore, ALND may be completely omitted because the prognosis remains unchanged despite SLNs being positive for micrometastases (<2 mm but >0.2 mm) (Galimberti et al. 2013; Maaskant-Braat et al. 2011). Some studies have explored this option in macrometastases (>2 mm) (Giuliano et al. 2011; Agresti et al. 2014; Donker et al. 2014), however it remains uncertain whether ALND is completely unnecessary. Thus, it is important to completely understand the anatomy and number of lymphatic routes and SLNs and their location.

A total of 480 cases clinically diagnosed with node-negative breast cancer underwent preoperative SLN CT-LG. Of these, 16 (6, previous ipsilateral breast surgery; 10, preoperative chemotherapy) were excluded from this study because Lizarraga et al. (2013) reported that SLNs were poorly visualized after surgery on the ipsilateral chest or axilla. Van der Ploeg et al. (2010) reported that, compared with untreated breasts, lymphatic flow in a previously treated breast occurs less often to the axilla and more often to nodes elsewhere, as far away as the contralateral side. In the present study, there was no lymphatic flow to the extra axilla, but five of the six surgery cases showed poor visualization. Previous reports also suggested that chemotherapy interferes with subsequent scintigraphy and lowers the SLN detection rate (Kuehn et al. 2013). Accordingly, 2 of the 10 cases that underwent preoperative chemotherapy showed poor visualization.

Thirty cases (6.5 %) were excluded from anatomical classification due to poor imaging, a proportion similar to in previous reports (Yamamoto et al. 2016; Takahashi et al. 2008). The causes of poor imaging can be classified into two categories: (1) the contrast agent does not flow into the lymphatic routes and lymph nodes, resulting in no capture; and (2) the contrast agent flows out from the lymphatic routes or lymph nodes before the scan. Probable reasons for obstruction of contrast agent include flow occlusion by tumor cells in the lymphatic routes and lymph nodes (Lehman et al. 2013). However, using the chi-squared test, there was no correlation between image quality and metastasis (*p* = 0.22). The flowing out of the contrast agent could be attributed to the agent being washed out before the scan, possibly due to increases in the number and diameter of lymphatic routes and the lymphatic network formed due to tumor-mediated lymphangiogenesis (Ran et al. 2010). Another possible reason for the flowing out of the contrast agent is the use of a water-soluble agent instead of an oil contrast one, such as ethyl ester of iodinated poppy seed oil fatty acid (Gómez et al. 2012). Although poor visualization can result from problems in the procedure, it is difficult to believe that these two are substantially related, because the injection of the contrast agent and scanning are sequentially and immediately performed. However, the reasons for such poor imaging should be investigated further.

Several injection techniques for SLN detection have been used to date, such as peritumoral or intranipple injection. In previous studies (Suga et al. 2003; Minohata et al. 2011), injection directly into the nipple was performed because it allows easy observation of morphological and anatomical features, such as the numbers of lymphatic routes and lymph nodes, and avoids use of image-guided injections of nonpalpable breast lesions. On the other hand, Suami et al. (2009b) stated that, compared with peritumoral injections, radioisotope intranipple injections alone may not detect some SLNs because numerous valves regulate the lymphatic flow and prevent its backflow. However, their findings are not necessarily applicable to the present study because of the variations between assessing dead and living bodies. Blumgart et al. (2011a) reported that lymphatic flow should be evaluated without peritumoral injections because peritumoral injections of radioisotope can cause a large region of breast tissue to contain radioactive tracer, thereby making observations less precise. Several other reports have stated that SLN identification is not affected by the injection site regardless of whether the injection is intranipple or peritumoral (Kern 2001; Caruso et al. 2014; Rosenow et al. 2012). Therefore, the present study used intranipple injection.

From the results of this study, single route/single SLN was the predominant category, while single route/multiple SLNs and multiple routes/multiple SLNs categories accounted for 10 % of cases; these results are similar to those reported by Yamamoto et al. (2016). The prevalence of multiple routes/single SLN was 6.0 % in the present study and 11 % in the report of Yamamoto et al., representing only a marginal difference.

In the present study, Yamamoto’s four categories of breast CT-LG were further subdivided based on branching patterns and the height at which the branching occurred. Such subdivision showed that lymphatic networks were complicated, and that CT-LG was useful in detecting such anatomical complexity.

Kutsuna (1968) and Kaneko (2001) reported cases where the lymph flowed into the parasternal lymph node, into the infraclavicular or supraclavicular lymph nodes without going through the axilla, and into the axilla of the contralateral side. There were no cases of lymph flow into the infraclavicular or supraclavicular lymph nodes without going through the axilla, or into the axilla of the contralateral side, in the present study. Lymphatic flow to the parasternal lymph node and to the supraclavicular lymph node via the axilla were observed.

According to the anatomical definition, the axilla refers to the indentation under the shoulder joint (Moore et al. 2014). The region of the axillary lymph nodes in breast cancer is wide and extends not only to the axillary locus, but also to the lateral chest and surrounding clavicle. In agreement with this fact, it was clearly shown that the axillary SLNs were widely distributed, and that a focus restricted only to the axillary locus could lead to potential oversight. To avoid such omissions, it is important to understand the location of SLNs. Therefore, CT-LG was used for easy classification of SLN location. While some SLNs can be identified using blood vessels and muscles as landmarks, the central axillary and pectoral lymph nodes, especially those distributed from the axillary region to the lateral chest, are difficult to locate. However, the ribs can be used to facilitate location of these lymph nodes; pectoral lymph nodes can be divided into the upper group (between the second and third ribs) and the lower group (between the fourth and sixth ribs) (Akiyama et al. 2015), whereas the central axillary lymph nodes are distributed above the upper group. Furthermore, since CT-LG was performed in the same posture as that during surgery, SLN location should not vary, which is considered very useful.

In conclusion, wide variation in the number of lymphatic routes and their branching patterns, number of SLNs and their locations, and the flow direction of SLNs was seen using CT-LG. CT-LG is useful for detecting lymphatic routes and SLNs in breast cancer and understanding SLN morphology, and can be easily implemented in settings with ordinary CT. Better understanding of the anatomical morphology of lymphatic routes and SLNs preoperatively is considered clinically very important and useful for treatment of breast cancer.
